# Transportation conditions of calves upon arrival at major livestock auction markets in Québec, Canada

**DOI:** 10.3168/jdsc.2023-0514

**Published:** 2024-03-29

**Authors:** Marianne Villettaz Robichaud, Marie-Pascale Morin, Gilles Fecteau, Sébastien Buczinski

**Affiliations:** 1 Département des sciences cliniques, Faculté de médecine vétérinaire, Université de Montréal, St-Hyacinthe, QC, J2S 2M2, Canada; 2 Regroupement pour un lait de qualité optimale, Op+lait, St-Hyacinthe, QC, J2S 2M2, Canada; 3 Bovine Health Research Group, GRESABO, Saint-Hyacinthe, QC, J2S 2M2, Canada; 4 Département des sciences cliniques, Faculté de médecine vétérinaire, Université de Montréal, St-Hyacinthe, QC, J2S 2M2, Canada; 5 Research chair in biosecurity of dairy production, Faculté de médecine vétérinaire, Université de Montréal, St-Hyacinthe, QC, J2S 2M2, Canada

## Abstract

The objective of this cross-sectional observational study was to describe the transport conditions of calves at the time of their arrival at the 2 major livestock auction markets in the province of Québec, Canada, and to identify characteristics that affect bedding cleanliness. A particular emphasis was placed on the transport environment of young dairy calves commonly being marketed for veal production. During 4 d per auction site (n = 2 sites), 2 d in summer and 2 d in winter, the descriptive characteristics including type of transports, number of calves per transport, separation from other transported animals, as well as presence of ventilation sources (e.g., open holes allowing natural ventilation), bedding, and bedding cleanliness, were determined. A total of 507 different transports were included, representing a total of 4,054 calves sold during these 8 d. The vast majority of calves (95% [n = 3,845]) were transported by commercially designed trailers (long commercial trailers (n = 358; 70.6% of all transport types), short commercial trailers (n = 62; 12.2%), or multideck trailers (n = 15; 3%). A minority of calves (5%) were either transported by homemade trailers (n = 30; 5.9% of transport) or other types of transports (n = 42; 8.3%). The presence of any ventilation source in the calves' transportation area was observed in 86% of transports and increased in summer versus winter (odds ratio: 2.75 [95% CI: 1.58–4.79]). Bedding was present in 96% of evaluated transports. The majority (68%) of calves' transport flooring area was considered clean, with less than 33% of the calves' area soiled with manure. The dirtiness of calves' transport flooring area was lower in winter than in summer (odds ratio = 0.63 [0.43–0.92]) and in site B than in site A (odds ratio = 0.57 [0.38–0.94]). This study gives interesting insight into transportation and unloading conditions of surplus calves in commercial auction markets.

## Highlights

• A large majority of calves were transported in commercially designed trailers.
• The specific area where calves were kept during transport was dirtier in summer than in winter and differed between auction sites.
• Despite being challenging, research in commercial setting conditions for calf transport is promising.

## Graphical Abstract

**Figure:**
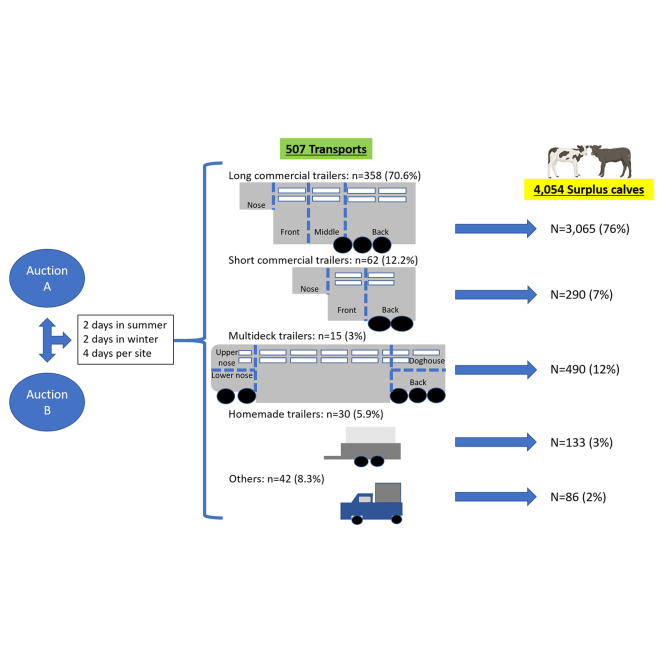
**Summary:** Little is known about the types of transport used to convey surplus calves from Québec dairy farms to auction markets. We described the types of transport used for surplus calves in 2 major auction markets during both summer and winter seasons. Assessments were conducted over 2 days for each season and at each auction. Additionally, we evaluated the cleanliness, the presence of ventilation, and the bedding in the areas where calves were kept during transport. The vast majority (95% of calves; n = 3,845) were transported in commercially designed trailers. A minority of calves (5%) were either transported in homemade trailers (n = 30; 5.9% of transport) or other types of transport (n = 42; 8.3%). We observed seasonal variations, with increased presence of ventilation sources and decreased cleanliness during the summer compared with the winter. Cleanliness also differed between the auction sites.

In the province of Québec, Canada, male calves and dairy crossbred calves born on dairy farms represent the main source of calves purchased for the production of veal and other types of beef. Calves are generally transported to be sold at the auction market at 1 to 2 wk of age corresponding to a typical BW of 45 to 54 kg in most calves (Buczinski et al., 2021). Transportation may involve long distances and may be associated with negative health impacts such as dehydration (Goetz et al., 2022), diarrhea (Goetz et al., 2023), or an increased risk of mortality (Boulton et al., 2020). Transporting newborn calves can be more challenging than older cattle because calves have an immature immune system and a lack of exposure to new environments (Roadknight et al., 2021). Transport stress can predispose calves to a high risk of morbidity and mortality. This may result in reduced mean daily gain during the growing phase (Roadknight et al., 2021). On a yearly basis, 100,000 to 130,000 calves are transiting through Québec auction markets, with almost 70% of calves in the 2 largest auction sites (Buczinski et al., 2021). However, little information is available on the characteristics of commercial transportation of these calves. It is, therefore, important to collect information of the transport conditions of young dairy calves to better understand this important component of surplus calves' journey in Québec. Most research on this topic arises from experimental transport conditions, which may differ from “real” conditions of transport (Roadknight et al., 2021). Thus, the overall objective of this project was to describe the transport conditions for Québec surplus calves in the livestock industry upon their arrival at 2 large auction markets, to identify characteristics that affect bedding cleanliness, and to determine seasonal variability.

The present study was part of a larger project on welfare and health on surplus dairy calves during the marketing process. This study was completed in accordance with the guidelines of the Institutional Animal Care Committee (CÉUA) of the Université de Montréal (CÉUA protocol #19-Rech-2015). A descriptive study of transport conditions during marketing of young dairy calves was conducted on the 2 largest livestock auction markets in Québec, between June 2019 and February 2020. Each auction market was visited 4 times, twice in summer 2019 and twice in winter 2020. We did not establish a predefined sample size for this descriptive study due to the absence of prior knowledge regarding young calves' transport types in Québec auction markets.

A single observer was responsible for evaluating the transport conditions and unloading of animals arriving at the auction markets for the entire study. Visual evaluation of trailers and transport conditions was performed in the large animal landing areas of the auction markets. The trailer type was categorized into 5 distinct groups: short commercial trailer (i.e., trailer lower flooring length is less than the length of 2 adult cattle placed one in front of the other), long commercial trailer (i.e., trailer lower flooring length is equal or longer than the length of 2 adult cattle in a single file), multideck trailers, homemade trailers (trailer not initially designed for transporting animals), as well as a variation of several types of box or pick-up grouped as “others.” This information is presented in Table 1, including details about the quantity and types of animals (calf or cow) being unloaded. All transports unloading at least one head of cattle (calf, heifer, cow, or bull) were evaluated (n = 650) in terms of type of trailer. For trailers carrying at least one calf (n = 507), additional information was collected. The information collected accounted for the fact that the auction market's unloading process could not be impaired. Therefore, data collection had to be performed quickly (<1 min after the animals were unloaded) before the departure of the trailer and clinical examination of the calves (Ramos et al., 2023). The specific location of the calves in the trailer was noted. Depending on the type of trailer, up to 5 different compartments allowing separation of the animals were distinguished: nose, deck, belly, back, and doghouse (González et al., 2012a). Ventilation was noted as a dichotomous variable focusing on the presence of any source of natural ventilation. The area of the trailer where calves were transported being either ventilated or not at the time of observation with the presence of ridges, opening, or open windows. No specific measurement of opening area was noted. Similarly, each transport carrying calves was classified as having or not having bedding in the area where calves were located. Dirtiness of the floor where the calves had been transported was scored on a scale of 1 to 3 (1 when <33% of the surface was visibly wet or soiled with manure, 2 when ≥33% but <66% of the surface was visibly wet or soiled, 3 when ≥66% of the surface was visibly wet or soiled). We chose this convenient trichotomous scale in the absence of a current consensus on transport area cleanliness assessment in livestock. Finally, it was also noted if a physical barrier was used to separate calves from other types of animals. The ambient temperature was obtained from the closest weather station from the auction site.

**Table 1:** Descriptive characteristics and comparison of transports arriving with young calves at 2 large Québec auction markets

Characteristic	Summer, n = 251			Winter, n = 256			*P*-value, season	*P*-value, site
	N	Site 1, n = 152	Site 2, n = 99	N	Site 1, n = 128	Site 2, n = 128		
Transport	251			256			0.29^1^	1.00^2^
Long trailer		99 (65%)	68 (69%)		98 (77%)	93 (73%)		
Short trailer		25 (16%)	12 (12%)		11 (8.6%)	14 (11%)		
Multideck trailer		2 (1.3%)	5 (5.1%)		3 (2.3%)	5 (3.9%)		
Homemade trailer		8 (5.3%)	7 (7.1%)		6 (4.7%)	9 (7.0%)		
Other		18 (12%)	7 (7.1%)		10 (7.8%)	7 (5.5%)		
Presence of ventilation	250			254			<0.001^1^	<0.001^2^
Absence		11 (7.3%)	9 (9.1%)		17 (13%)	34 (27%)		
Presence		140 (93%)	90 (91%)		111 (87%)	92 (73%)		
Presence of bedding	249			255			0.47^1^	0.21^2^
Absence		8 (5.3%)	3 (3.1%)		3 (2.3%)	4 (3.1%)		
Presence		143 (95%)	95 (97%)		125 (98%)	123 (97%)		
Dirtiness	245			252			0.03^1^	<0.001^2^
≤33% of soiled area		89 (60%)	65 (68%)		84 (66%)	104 (83%)		
>33 to ≤66% of soiled area		34 (23%)	17 (18%)		24 (19%)	8 (6.4%)		
>66% of soiled area		26 (17%)	14 (15%)		19 (15%)	13 (10%)		
Number of calves per transport, median (IQR)	251	5 (3, 10)	7 (3, 15)	256	5 (3, 9)	5 (2, 9)	0.06^3^	0.32^3^
Number of cows per transport, median (IQR)	251	2 (0, 5)	3 (0, 6)	256	4 (1, 7)	4 (1, 6)	<0.001^3^	0.32^3^

1 Cochran-Mantel-Haenszel test.

2 Breslow-day test for testing OR homogeneity.

3 Kruskall-Wallis test.

Although it was impossible to determine the exact time spent in the transportation in this study, we previously estimated in Ramos et al. (2023) that the median distance traveled by these calves was 57 km (interquartile range [**IQR**; 32.4–106.2 km] ranging from 0.9 to 581.2 km), using the individual identification number of the calves, which is associated with farm geographical coordinates in auction market database. It was not possible to link specific calves' findings with transport conditions and unloading technique due to the rapid unloading flow.

Statistical analyses were performed using R software (R Development Core Team, 2020). Descriptive statistics concerning the number of transported animals (calves and cows in transports including at least 1 calf) were reported as median and IQR. Descriptive results to the location of the calves in transport were also indicated. A Cochran-Mantel-Haenszel test was used to determine whether the season affects the presence of ventilation or bedding (dichotomous outcomes) in the trailer when adjusting for auction site as a potential confounder. Breslow-Day test was performed to determine odds ratio (**OR**) homogeneity in both auction sites. The associations between season, auction site, and number of calves and cows transported per transport were tested using nonparametric ANOVA (Kruskall-Wallis test). The association between the ventilation as well as cleanliness with transport type were tested with a chi-squared test with Bonferroni adjusted post hoc comparison when the global test was significant.

For calves' bedding dirtiness (trichotomous variable), a multivariable ordinal logistic regression was performed. All variables of interests (number of calves transported within the trailer, transport type, season, and auction sites) were initially entered in a full model and a manual backward selection was performed until all remaining variables were significant at the *P* < 0.05 thresholds, except auction site, which was forced in the model. Collinearity and confounders were checked using variance inflation factor and change or more than 20% of the regression coefficient, respectively. Significance was set at *P* < 0.05 for all the analyses. Model fit was assessed using the specific Lipsitz test (Lipsitz et al., 1996) for ordinal regression models (*P* > 0.05 indicates that the model satisfies the proportional odds assumption).

The study took place on June 26 and July 1, 3, and 8, 2019, for the summer data collection and on February 10, 12, 22, and 24, 2020, for the winter period. The maximal (minimal) temperatures recorded in the auction sites were +27.5°C (+17.3°C), +26.5°C (+9.5°C) for summer and +1.0°C (−12.0°C), +1.0°C (−3.5°C) for the winter sampling in site A. For site B, +26.1°C (+12.4°C), +28.0°C (+13.8°C) were recorded for the summer sampling and −3.0°C (−14.0°C), +8.5°C (−28.5°C) for the winter sampling days. In total, the unloading of animals from 650 transports was observed during the study period (366 at auction A and 284 at auction B). We only focused our observations on the 507 transports that included at least one calf. Transports without calves were not included. A total of 280 in auction A (152 in summer and 128 in winter) versus 227 in auction B (99 in summer and 128 in winter) were included, as detailed in Table 1. This represented a total of 4,054 calves (2,053 for site A and 2,001 for site B). The remaining transports unloading on these days (n = 143; 28%) did not transport any calves. Most transports with calves consisted of long commercial trailers (n = 358; 70.6%), followed by short commercial trailers (n = 62; 12.2%), multideck trailers (n = 15; 3%), homemade trailers (n = 30; 5.9%), and other types of transports (n = 42; 8.3%), as represented in Figure 1. Long commercial trailers transported the majority of calves (n = 3,065; 75.6%), followed by multideck transports (n = 490; 12.1%), and short commercial trailers (n = 290; 7.2%). Homemade trailers as well as other types of transports, represented a small number of transported calves with 183 (3%) and 86 (2%) calves, respectively. We have represented the distribution of all transports, including the number of calves per transport and the total number of transported calves by auction site in Figure 1 and Table 1. A large majority of transports (485 of 507; 95.7%) had isolated calves from other animals (n = 377) or only transported calves (n = 108). Calves were always isolated in short commercial trailers, multideck, and other types of transport (e.g., box, pickup). When calves were not isolated from other types of animals (n = 22; 4.3%), they were in direct contact with small ruminants (n = 12), older calves (n = 4), dairy heifer (n = 3), adult cows (n = 3), or young bulls (n = 2). For calves transported in long, short commercial, and multideck trailers, it was possible to determine their location during the transportation. For long commercial trailers, calves were present in the front (n = 293; 81.8%), the nose (trailer space above the truck attachment; n = 15; 4.2%), the middle (n = 8; 2.2%), or simply in the back (n = 46; 12.9%). A total of 4 (1.1%) long commercial trailers had calves in more than one area. For short commercial trailers, calves were present in the front (n = 33; 53.2%), the nose (n = 2; 3.2%), or simply in the back (n = 27; 43.6%). In multideck trailers, calves were present in the upper nose (n = 8; 53.3%), lower nose (n = 1; 6.7%), deck (n = 5; 33.3%), back (n = 5; 33.3%), or in the doghouse (n = 5; 33.3%). In 9 (60%) of the multideck trailers, calves were positioned in at least 2 locations.

**Figure 1:**
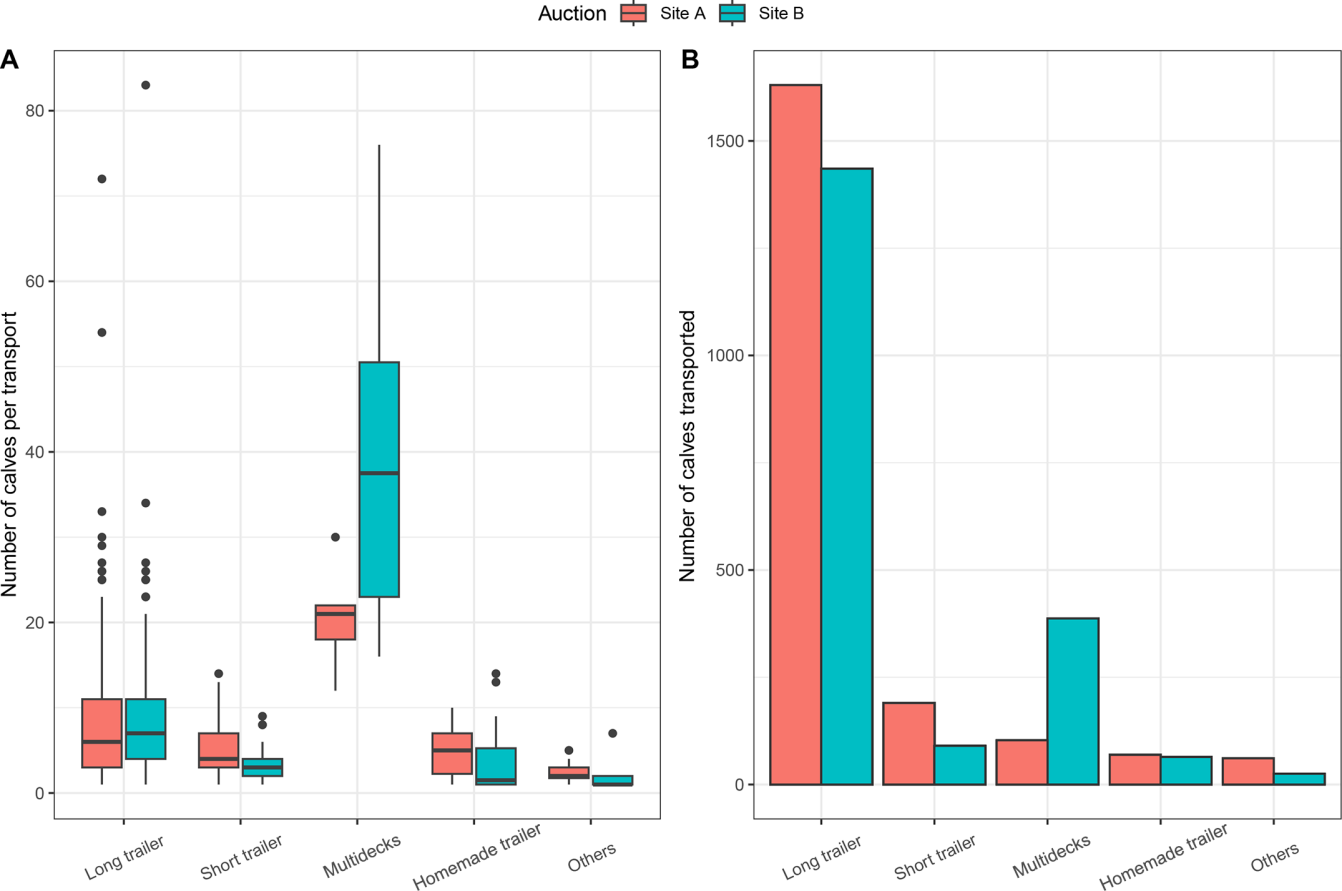
Distribution of the number of calves per transport by type of trailer and total number of calves transported by specific trailer type in 2 Québec auction markets during 8 different auction days. Panel A shows the number of calves unloaded per transport by the different trailer types in both auction sites (A and B), and panel B shows the total number of calves unloaded from the different types of trailers in the 2 auctions. The lower and upper hinges represent the IQR. The whiskers extend from the hinges to the value of up to 1.5 × IQR (the same for lower whiskers). The dots represent values exceeding this previous threshold.

The majority of transports (85.9%) had at least one ventilation source in the specific calves' laying area during transport (433 of 504 recorded data). No difference was observed between transport type and level of cleanliness (*P* = 0.25). In contrast, transport type was associated with the presence of ventilation (*P* < 0.001), which was more often absent in homemade (proportion of 8/19) and other types of transports (21/42) than in long trailers (30/355), short trailers (8/66), or multideck trailers (0/15). After adjusting for auction site, the odds of the presence of any ventilation area increased in summer compared with winter, with an OR of 2.75 (95% CI: 1.58–4.79; *P_CMH_* < 0.001, *P_Breslow-day test_* < 0.001). The odds of presence of any ventilation area was increased in summer when compared with winter, after adjusting for auction site (OR: 2.75 [95% CI: 1.58–4.79]; *P_CMH_* < 0.001, *P_Breslow-day test_* < 0.001). The presence of bedding was observed in 486 of 504 transports (96.4%) and was not associated with the season (OR: 0.618 [0.232–1.64]; *P_CMH_* = 0.47) or auction site (*P_Breslow-day test_* = 0.21). The proportion of transports with <33% of wet or dirty flooring surface where calves were kept was 68.8% (342 out of 497 transports where information could be assessed), and 18.1% of transports could be considered dirty (n = 72) with more than 66% of the surface being wet or dirty (see Figure 2). The season and the site were associated with increased odds of dirtiness. The calves' transport flooring area was less dirty in winter than in summer (OR = 0.430 [0.381–0.631]; *P* = 0.018) and in site B than in site A (OR = 0.835 [0.566–923]; *P* = 0.005) in the final multivariable ordinal logistic regression (Supplemental File S1; see Notes). The Lipsitz test indicated a good model fit (*P* = 0.16).

**Figure 2:**
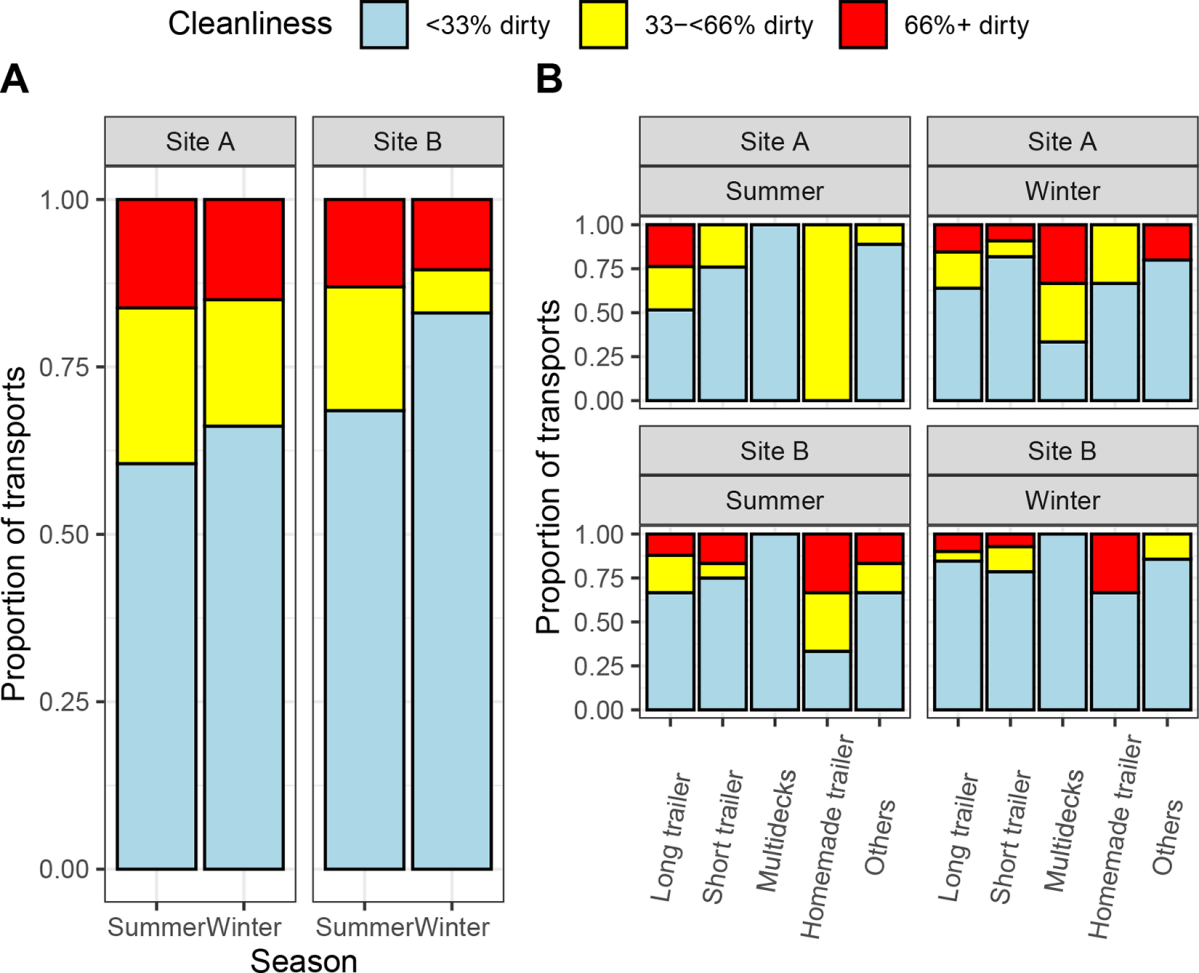
Proportion of dirtiness categories in areas where calves were transported by auction sites and season (panel A) and when considering the type of trailer (panel B). The data were collected during 2 d in the summer and 2 d in the winter for each auction site.

During this study, we noticed that the majority (75.6%) of transported calves were in long commercial trailers, whereas a minority were in other types of trailers. Other animals, generally cows, were transported in the trailers transporting calves, but were most often separated from these other types of animals. Approximately 20% of the assessed transports were considered dirty with more than two-thirds of the calves' laying area being judged as wet or dirty at unloading. The general requirement for transporting animals in Canada requires, among other specifications, the use of a specially designed container or vehicle for animal transportation, to provide adequate space, adequate ventilation, and protection in case of extreme weather, as well as providing a nonslip area (Canadian Food Inspection Agency, 2020). We could not assess specific characteristics of bedding (e.g., bedding depth, moisture content) that could be associated with increased risk of slipping, other than apparent cleanliness observed during unloading. The ventilation was also qualitatively assessed (presence or absence) but could not be quantitatively measured. However, we think that the vast majority of transports assessed during this study meet Canadian regulation. The current study shows that a large proportion (85.6%) of transports were specially designed trailers, whereas homemade trailers and other types of transport procedures were uncommonly used but had lower presence of source of ventilation than long, short, and multideck trailers. It is unknown if the calves' welfare during transportation is different in the various types of transports. We hypothesized, even if we cannot prove it, that most of these nonspecially designed transports could represent individual farms close to the auction sites who directly transport their calves without a professional livestock transportation service. Future research on calves' transportation should not underestimate the importance of these alternative ways to transport calves (homemade trailers and box vs. trailers primarily designed for calf transportation), especially when transport is performed by people with less training and experience.

Gathering data from commercial livestock transport of young calves is a challenge as recently reviewed by Roadknight et al. (2021). The process of traveling from a farm to another, including loading the animals to arrive on time at the auction market, makes research projects challenging. Determination of calves' transportation characteristics and impact on their health parameters was not possible in this study but deserves further work. In a British Columbia study, surplus calves evaluated before and after transport to an auction market or to one calf grower had greater odds of being depressed when suffering from failure of transfer of passive immunity, but no information was available on the type and characteristics of transports involved (Wilson et al., 2020). In an experimental Dutch study with either 6 or 18 h traveling duration, 3-wk-old calves transported in conditioned trucks had lower umbilical infection problems during the early feeding period in veal farms than calves transported in open trucks (Marcato et al., 2020). Abnormal physical findings are commonly observed in Canadian young calves transported to auction market (Marquou et al., 2019), or upon arrival at the veal unit (Scott et al., 2019). However, it remains partially unknown what proportion of abnormalities are caused or worsened by different transport characteristics.

The current study is a first step aiming to gather a realistic portrait of the young calves' transportation conditions to auction market. Adequate bedding can improve transportation conditions, as it was associated with lower serum creatine kinase concentration (a marker of muscle injury) observed after transporting Australian bob-calves (Jongman and Butler, 2014). This improvement could be attributed to the decreasing muscle compression during lying or preventing muscle injury associated with accidental falling. Dry and abundant bedding will also help thermoregulation of the young calves, especially during cold periods (Roland et al., 2016). The relationship between bedding dirtiness or dryness and calves' welfare during transport still needs to be explored, but a slippery surface may be associated with higher risk of muscle damage and bruising during transport. Moreover, the presence of a dirty surface may increase the risk of exposure to fecal pathogens or decreased air quality during transportation when the calves are lying and could therefore be a possible risk factor for enteric or respiratory disease, or antimicrobial-resistant pathogens after transportation (EFSA Panel on Biological Hazards et al., 2022). Young calves generally lie down during the transportation, in contrast to adult cattle, who generally stand (Cockram and Spence, 2012). Therefore, stocking density during transport and bedding thickness are also important factors that could affect the calves' welfare, which were not possible to measure in this study.

Transport duration or distance is another important aspect that can also be associated with the presence of more contaminated bedding. In a New Zealand study by Boulton et al. (2020) on case-control analysis of calves dead on arrival or during lairage, each additional 1 h of transport from the farm to the slaughter plant increased the odds of dying (OR = 1.45; 95% CI: 1.18–1.76). However, it was not possible to associate the mean distance traveled with transport area cleanliness.

This study aimed to describe the basic characteristics of transports involving young calves arriving at 2 large auction markets in both summer and winter. The study was limited to 2 visits per auction per season. While we believe that the results are representative of what is routinely observed in these auctions, based on authors' experience and discussions with various stakeholders, the limited number of observation-days may be a limitation of this study. Moreover, because transport regulation and marketing characteristics depend on local regulation and geographical particularities, extrapolation to other settings should be done with caution.

Designing research projects on livestock commercial transport conditions remains an important challenge to the involvement of numerous actors and the wide variety of trailers, environments, and journeys the animals may undertake. This study represents a first step to understand the commercial transport conditions on young calves in Québec, Canada. Most calves were transported in commercial trailers, along with other animals, and were provided with ventilation and bedding during transport. The lack of cleanliness of the trailer areas where calves were located in the transport may indicate a need for adjustment in bedding and ventilation management during transport.
